# Evidence-based directed acyclic graphs for perinatal pharmacoepidemiologic studies in rheumatology: a structured approach for development and implementation in administrative health data

**DOI:** 10.3389/fepid.2026.1737016

**Published:** 2026-03-10

**Authors:** Vienna Cheng, Neda Amiri, Vicki Cheng, Jacquelyn J. Cragg, Laurie Proulx, Mary A. De Vera

**Affiliations:** 1 Faculty of Pharmaceutical Sciences, University of British Columbia, Vancouver, BC, Canada; 2 Collaboration for Outcomes Research and Evaluation, Vancouver, BC, Canada; 3 Arthritis Research Canada, Vancouver, BC, Canada; 4 Division of Rheumatology, Department of Medicine, Faculty of Medicine, University of British Columbia, Vancouver, BC, Canada; 5 Pregnancy and Rheumatic Diseases Clinic (PReDICT), Mary Pack Arthritis Centre, Vancouver, BC, Canada; 6 International Collaboration on Repair Discoveries (ICORD), University of British Columbia, Vancouver, BC, Canada; 7 Canadian Arthritis Patient Alliance, Ottawa, ON, Canada; 8 Patient Author, Ottawa, ON, Canada; 9 Centre for Health Evaluation and Outcome Sciences, Vancouver, BC, Canada

**Keywords:** directed acyclic graphs, disease-modifying antirheumatic drugs, methodology, pharmacoepidemiology, pregnancy, rheumatic disease, rheumatology

## Abstract

**Background:**

Evidence-based Directed Acyclic Graphs (DAGs) are effective tools to comprehensively visualize complex causal and biasing pathways in pharmacoepidemiologic research in rheumatology. This paper outlines the process of developing and implementing a DAG, using a cohort study evaluating the impact of targeted synthetic disease-modifying antirheumatic drugs (tsDMARDs) on congenital anomalies as a case example. We include a discussion of how factors would be operationalized into variables in administrative data within the case example.

**Methods:**

DAG Development involved: 1) identifying exposure and outcome, 2) identifying factors affecting the exposure, 3) identifying factors affecting the outcome, 4) identifying factors affecting both the exposure *and* outcome, 5) ascertaining relationships between factors, and lastly, 6) finalizing the DAG in DAGitty v3.1.

**Results:**

The final DAG for our case example on evaluating the association between tsDMARDs and congenital anomalies consisted of 21 nodes (points in the diagram representing factors such as exposures, outcomes, confounders, or mediators): 1 affecting the exposure, 12 affecting the outcome, 7 on the biasing pathways, and 1 mediator (maternal infection) on the exposure-outcome pathway. One minimally sufficient adjustment set was identified to inform confounder adjustment in a multivariable model, consisting of: concomitant conventional synthetic DMARDs, rheumatic disease activity, and maternal demographics (i.e., age, place of residence, race/ethnicity). Implications for implementing this DAG in a study using administrative health data include comprehensively revealing confounders to be adjusted for.

**Conclusions:**

Our systematic approach to developing a DAG is particularly valuable for improving study designs in the growing field of perinatal pharmacoepidemiology in rheumatology, where there is a critical need for robust perinatal data on novel arthritis medications.

## Introduction

As new pharmacotherapies continue to enter the market, the demand for robust evidence on the safety and risks of medications during pregnancy is growing. Perinatal pharmacoepidemiologic studies allow researchers to understand how perinatal drug exposures impact maternal, fetal, and neonatal health outcomes (1). As pregnant women are often excluded from clinical trials due to reasons such as randomization challenges and ethical concerns, much of perinatal research relies on observational studies (1). However, perinatal pharmacoepidemiologic studies are often complicated by methodological challenges, such as confounding by indication and other biases due to the complex relationship between exposures and outcomes (1).

Evidence-based causal Directed Acyclic Graphs (DAGs) is a powerful tool for visualizing existing and potential biasing pathways, including causal factors such as confounders and mediators, between drug exposures and outcomes (2). Causal DAGs allow researchers to detect biases and inform development of robust analytical epidemiological models (3, 4). This is done by depicting established or probabilistic associations between factors in the form of nodes and arrows (3). Nodes represent factors, whereas arrows represent directionality in the causal pathway between factors, suggesting a relationship. In particular, incorporating DAGs into the design of perinatal pharmacoepidemiologic studies enables researchers to identify biasing pathways [i.e., paths that lead to spurious associations between variables (2)] and determine minimally sufficient adjustment sets of confounders [i.e., the smallest group of variables that, when controlled for, eliminates bias from confounding, and allows for a valid estimation of the total causal effect between the exposure and outcome (5)], thereby enhancing the rigor and validity of studies (4, 6). For the purposes of this study, we use the term *DAG* to refer specifically to *causal DAGs*.

In health sciences research (e.g., pulmonary, cardiovascular), DAGs have been increasingly used to improve study design and interpretation by identifying confounding, selection, and other biases in observational studies (7). While previous studies have provided guidance and tutorials on definitions of terminology and on how to develop and apply DAGs in clinical research (8, 9), our paper details a set of structured steps for developing an evidence-based DAG using a relevant case example grounded in perinatal pharmacoepidemiology. This case example illustrates how evidence-based DAGs can be used to visualize causal pathways and identify appropriate confounder adjustment sets as part of the study design process when investigating a specific research question. We further discuss how to operationalize factors into variables, including strategies for addressing variables unavailable in the data, illustrated using administrative health data as an example. We acknowledge that alternative data sources may contain different variables; thus, we encourage researchers to adapt the operationalization of factors in their DAG according to variables available in their specific data sources. For purposes of this paper and the DAG development process, a *factor* refers to the conceptual construct of interest (e.g., sex, age), whereas a *variable* refers to the corresponding operationalized analytic concept included in a statistical model.

Perinatal pharmacoepidemiologic studies leverage a range of data sources to evaluate drug safety, effectiveness and utilization. These sources include administrative health databases, medical records, registries and primary data collection (10). Of relevance to our case example, administrative health databases provide longitudinal, population-based data that improve generalizability; however, they have limitations on variable availability (e.g., race/ethnicity, disease activity) and potential misclassifications in diagnosis coding (10). With growing therapies and calls for perinatal evidence, there is an increasing need to leverage these data and address associated limitations using tools such as DAGs.

**Figure 1:**
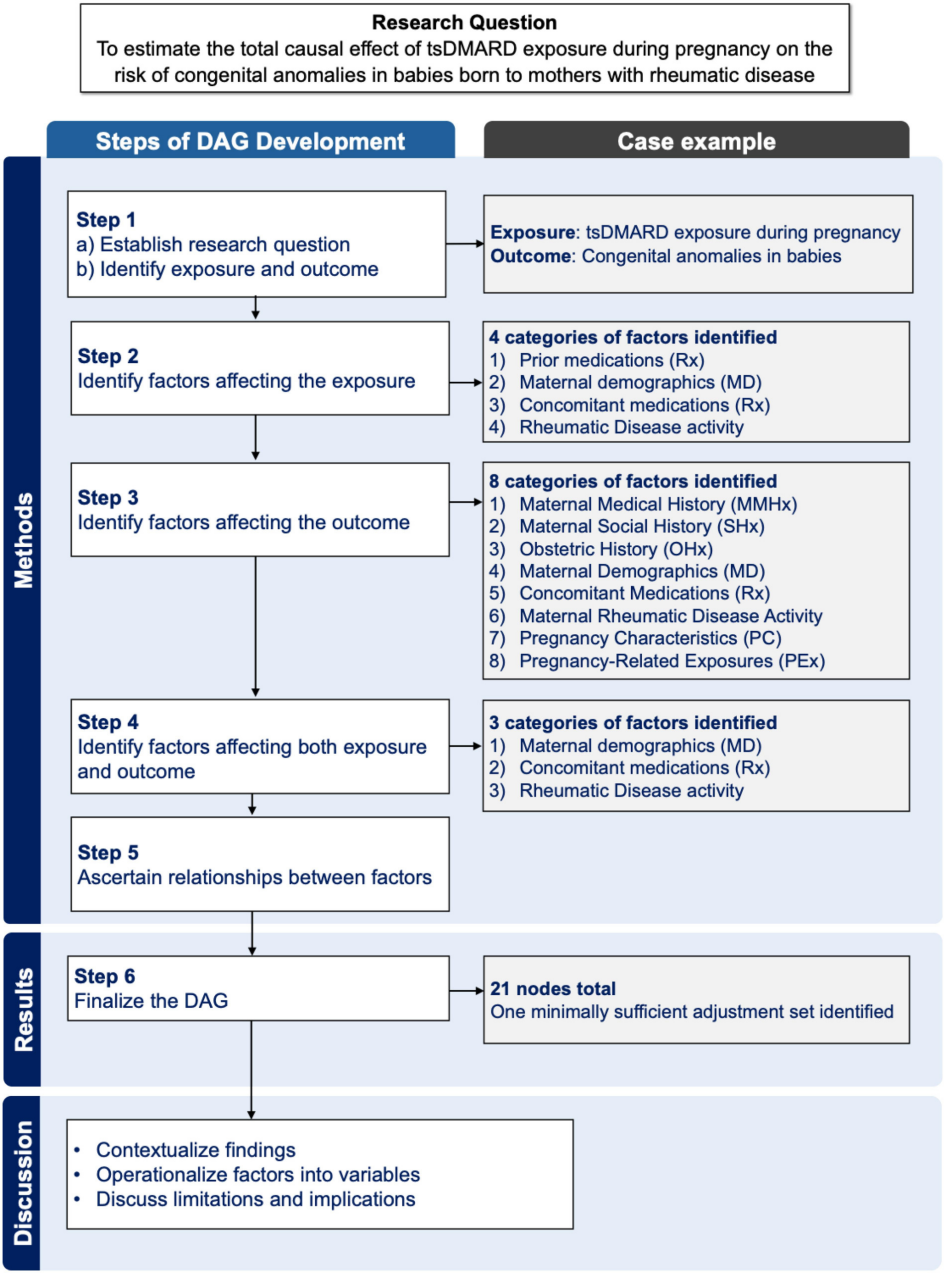
Schematic overview of the stepwise process of DAG development and corresponding results from the case example. Abbreviations for category names: MD, maternal demographics; MMHx, maternal medical history; OHx, obstetric history; PC, pregnancy characteristics; PEx, pregnancy-related exposure; Rx, medications; SHx, Social history.

To guide the development of the evidence-based DAG, we established a multi-disciplinary team of experts in rheumatology, causal inference, pharmacotherapy, perinatal epidemiology and a patient research partner with lived experience. Our DAG was developed through a 6-step process: 1) identifying exposure and outcome, 2) identifying factors affecting the exposure, 3) identifying factors affecting the outcome, 4) identifying factors affecting both the exposure and outcome, 5) ascertaining relationships between factors, and lastly, 6) finalizing the DAG. We have included a schematic overview of the DAG development process and corresponding results from our case example in Figure 1. Throughout these steps, we drew from prior literature, coupled with discussions between the team.

We chose to use DAGitty v3.1 as it is an accessible, free web-based application, and throughout our description of steps in developing a DAG, we also define concepts, terms and notations used specifically by this application (11). While we acknowledge that alternative tools exist to draw a DAG, the underlying concepts of causal diagrams that we discuss remain applicable regardless of the software used. Our use of DAGitty allowed us to provide a clear, reproducible process, including a model code (Supplementary File 1), that other researchers can adapt into their future studies.

### Case example: developing a causal DAG for a perinatal pharmacoepidemiologic study in rheumatology

As a case example, we developed a causal DAG as part of the design of a perinatal pharmacoepidemiologic study in rheumatology. Our research question aims to estimate the total causal effect of targeted synthetic disease-modifying antirheumatic drug (tsDMARD) exposure during pregnancy on the risk of congenital anomalies in babies born to mothers with rheumatic disease. This case example is particularly relevant in rheumatology given the limited evidence on the perinatal impacts of tsDMARDs, a new but effective treatment option for rheumatic disease (12). With respect to exposure, tsDMARDs are of particular concern due to their small molecular size, which allows them to potentially cross the placenta and affect the developing fetus (13). With respect to outcome, congenital anomalies are of particular concern in perinatal pharmacoepidemiologic studies and involve many factors that may influence their development (14). In pregnant females with rheumatic disease, the impact of tsDMARD exposure on congenital anomalies is a relationship that has yet to be explored in the literature (12), and serves as an ideal case example for demonstrating the application of DAGs to identify biasing pathways and inform confounder adjustment. In this paper, we describe a structured, step-by-step process for developing and implementing a causal DAG, with implications for informing future perinatal pharmacoepidemiologic research in rheumatology and beyond.

## Methods

### Identifying exposure and outcome

**Figure 2:**
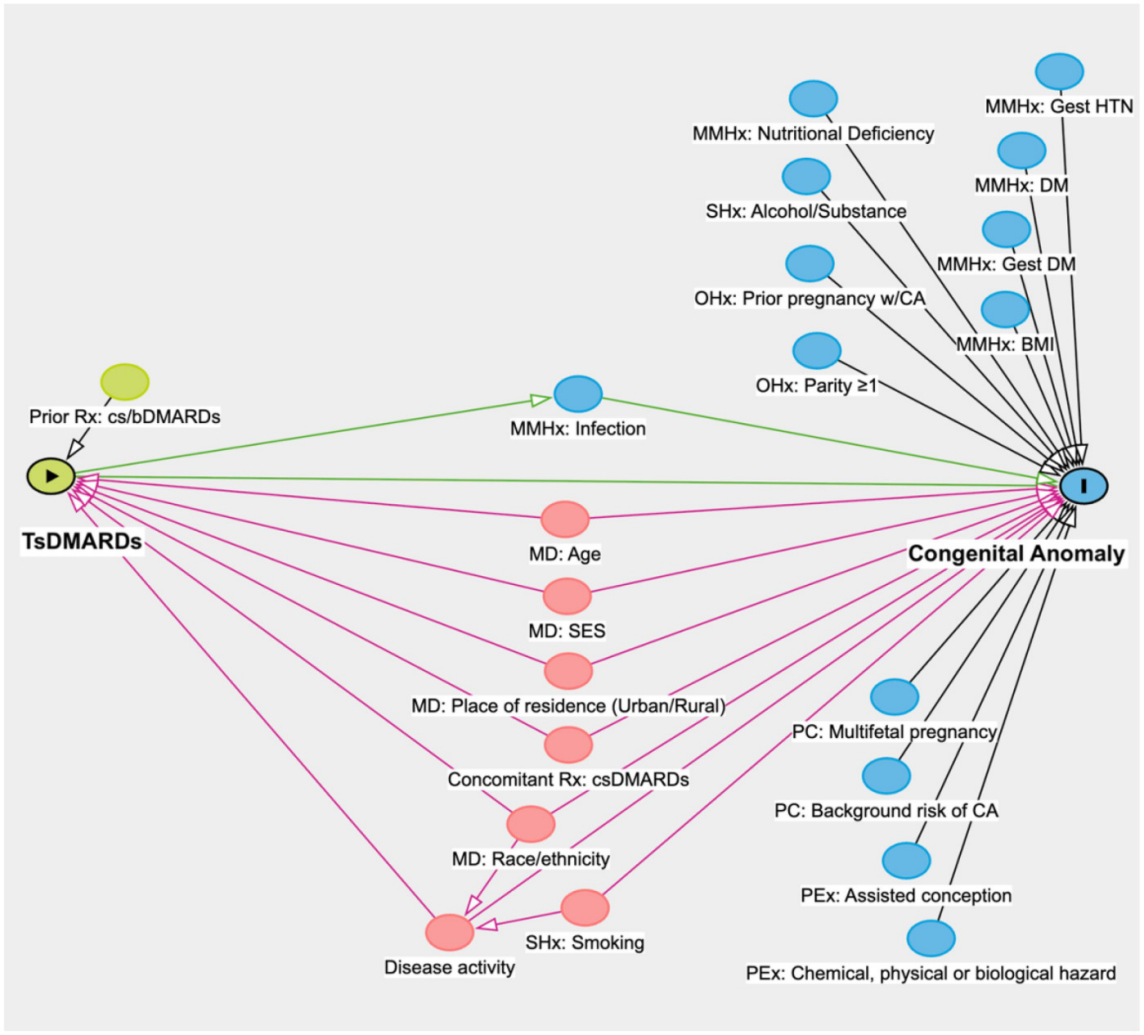
Directed acyclic graph (DAG) to examine the relationship between in-utero exposure of targeted synthetic disease-modifying antirheumatic drugs (tsDMARDs) and congenital anomaly. Abbreviations for category names: MD, maternal demographics; MMHx, maternal medical history; OHx, obstetric history; PC, pregnancy characteristics; PEx, Pregnancy-related exposure; Rx, medications; SHx, Social history. Other abbreviations: BMI, body mass index; CA, congenital anomaly; cs/bDMARDs, conventional synthetic/biologic disease-modifying antirheumatic drugs; DM, diabetes mellitus; Gest, gestational; HTN, hypertension; SES, socioeconomic status.

Our research question is to estimate the total causal effect of tsDMARD exposure during pregnancy on the risk of congenital anomalies in babies born to mothers with rheumatic disease. Following established frameworks for constructing causal diagrams (3, 15), we first defined the key components of our research question: the exposure and outcome. The exposure of interest was exposure to tsDMARDs during pregnancy. The outcome was congenital anomalies in the newborn. To illustrate this hypothesized causal relationship, a green node with a black triangle labelled “tsDMARDs” was created as the exposure and a blue node with an “I” labelled “congenital anomaly” was created for the outcome, connected by an arrow drawn directly from the exposure to the outcome node (Figure 2). Of note, a key consideration in this relationship is selection bias, as our outcome conditions on the delivery of a live birth. Selection bias on live birth, or live birth bias, occurs when analyses are restricted to pregnancies that result in live births, which excludes pregnancies resulting in miscarriage, stillbirth or termination. This can affect the external validity of the casual effect and distort the observed association between tsDMARD exposure and congenital anomalies. Future studies should consider sensitivity analyses to account for this bias. We identified “Maternal Medical History: Infection” (blue node) as a mediator, since tsDMARDs—particularly Janus kinase inhibitors—are immunomodulators that increase infection risk in the mother, which in turn may contribute to the development of congenital anomalies in the baby (14, 16).

### Identifying factors affecting the exposure

We then identified factors that could directly affect tsDMARD exposure. In DAGitty, these are referred to as “ancestors” and are represented by green nodes with unidirectional arrows pointing towards the exposure (Supplementary Figure 1). We identified the following four categories of factors that could affect a rheumatic disease patient's initiation of tsDMARD treatment: 1) Prior medications (Rx) [i.e., conventional synthetic (cs)/biologic (b)DMARDs (17)], 2) Maternal demographics (MD) [i.e., age (18), place of residence (19), race/ethnicity (18), socioeconomic (SES) status (20)], 3) Concomitant medications (Rx): csDMARDs (21), and 4) rheumatic Disease activity. Prior medications (Rx): cs/bDMARDs was added because it is common for patients to be trialed on cs/bDMARDs prior to tsDMARDs (22, 23).

We drew an arrow from “Maternal demographics: Age” to the exposure because compared to individuals over 65 years old, individuals under 40 years old [adjusted relative risk (aRR): 2.41, 95% confidence interval (CI): 1.83–3.19, *p* < 0.0001] and those aged 40–65 years (aRR: 1.63, 95% CI: 1.34–1.98, *p* < 0.0001) are more likely to be initiated on a biologic or tsDMARD (18). We added “Maternal demographics: SES” because a mother's SES can influence a number of factors including access to healthcare (20), drug affordability, health-seeking behaviors, and health literacy. We added “Maternal demographics: Place of residence” because in the absence of studies on tsDMARDs, prior studies on biologic DMARDs showed that patients with rheumatoid arthritis that live in rural residence have a higher likelihood of biologic drug initiation [hazard ratio (HR): 1.96, 95% CI: 1.28–2.99] (19). Next, “Concomitant medication: csDMARDs” was added because combination therapy with csDMARDs [e.g., methotrexate, a known abortifacient and teratogen (24)] and tsDMARDs have superior efficacy to csDMARD monotherapy, particularly in patients with rheumatoid arthritis (21). “Maternal demographics: Race/ethnicity” was added because literature has also revealed an association between race/ethnicity and tsDMARD initiation. Particularly, Russell et al. found that Asian individuals were 48% less likely than White individuals to be initiated on biologic and tsDMARDs (aRR: 0.52, 95% CI: 0.36–0.76, *p* = 0.0007) (18). Additionally, Black individuals were 54% more likely than White individuals to be initiated on biologics and tsDMARDs (RR: 1.54, 95% CI: 1.10–2.16, *p* = 0.012), though this was believed to be partially attributed to greater baseline disease severity (18). Lastly, rheumatic “Disease activity” was added as a factor because the higher the disease activity, the more likely one may be initiated on a biologic or tsDMARD (22, 23).

### Identifying factors affecting the outcome

We also identified factors that could directly affect our outcome of congenital anomalies. These are referred to as “ancestors” in DAGitty and represented by blue nodes with unidirectional arrows pointing towards our outcome (Supplementary Figure 2). Eight categories of risk factors for congenital anomalies were identified, listed below (Figure 2): 1) Maternal medical history (MMHx) [i.e., gestational hypertension (HTN) (25), diabetes mellitus (DM) (26), gestational DM (27), body mass index (BMI) (14), nutritional deficiency (26), infection (e.g., viral infection) (14)], 2) Maternal social history (SHx) [i.e., alcohol/substance use (26), cigarette smoking (26)], 3) Obstetric history (OHx) [i.e., prior pregnancy with congenital anomaly (CA) (28), parity ≥1 (29)], 4) Maternal demographics (MD) [i.e., age (29, 30), SES (14), place of residence (urban/rural) (31, 32)], race/ethnicity (33), 5) Concomitant medications (Rx) [i.e., csDMARDs (34)], 6) Maternal rheumatic Disease activity (35), 7) Pregnancy characteristics (PC) [i.e., multifetal pregnancy (36), background risk of congenital anomalies], and 8) Pregnancy-related exposures (PEx) [i.e., assisted conception (29); chemical, physical and biological hazard exposure (29)].

“Maternal demographics: Age” was included because the prevalence of babies born with congenital anomalies is higher among mothers <20 and ≥40 years old (30). “Maternal demographics: SES” was included because low neighborhood SES has been associated with congenital anomalies such as cleft palate [pooled odds ratio (OR): 1.22, 95% CI: 1.10–1.36] (14). “Maternal demographics: Place of residence” was added because a lower prevalence of congenital anomalies has been observed in rural compared to urban counties [Prevalence ratio (PR): 0.88, 95% CI: 0.87–0.89] (32). In terms of concomitant medications, glucocorticoid drugs were not included as a factor because non-fluorinated glucocorticoids (i.e., prednisone, which is most commonly used in rheumatic diseases) are readily metabolized by the placenta, and have not been associated with congenital anomalies (37). “Maternal demographics: Race/ethnicity” was added because there is a significantly greater prevalence of congenital anomalies in babies of populations such as non-Hispanic Black women (PR: 2.76, 95% CI: 2.16–3.53) (33). Maternal rheumatic “Disease activity” was added because rheumatic diseases such as rheumatoid arthritis have been associated with increased risk of congenital anomalies (OR: 1.24, 95% CI: 1.13–1.37, *I*^2^ = 42.3%) (35). “Pregnancy characteristics” were added because every pregnancy comes with a background risk of congenital anomalies (26), including those resulting from unknown causes (38) and carrying a multifetal pregnancy (36). Lastly, “Pregnancy-related exposures” such as assisted conception and hazard exposure have been associated with increased risk of congenital anomalies (29).

### Identifying factors affecting both exposure and outcome

From the DAG, we identified six factors that directly affect both the exposure *and* outcome, depicted by red nodes to signify ancestors (nodes that affect another node) of the exposure *and* outcome: Maternal demographics of 1) age, 2) place of residence (urban/rural), 3) race/ethnicity, 4) SES; Concomitant medication of 5) csDMARDs; and 6) Maternal rheumatic disease activity.

### Ascertaining relationships between factors

Relationships between factors were indicated as red arrows in the DAG, forming the biasing paths of this exposure-outcome relationship. We drew an arrow from “Maternal demographics: Race/ethnicity” towards rheumatic “Disease activity” because literature suggests higher disease activity and inflammatory burden in Latinx, Asian and Black individuals, compared to White individuals with rheumatoid arthritis (39). We also drew an arrow from “Social history: Smoking” to rheumatic “Disease activity” because cigarette smoking is associated with higher rheumatic disease activity (40). Altogether, these red arrows form the biasing paths of the exposure-outcome relationship. Of note, the overall goal of our DAG was to visualize causal pathways between the exposure and outcome by including only factors with direct associations. While we acknowledge that additional relationships, such as the association between SES and nutritional deficiency, may exist, we chose to not to include indirect pathways that do not directly confound or mediate the relationship between the exposure and outcome. Our focus remained on capturing the main causal pathways between the exposure and outcome to ensure clarity and interpretability, while acknowledging that additional complexities may exist.

## Results

### Finalizing the DAG

In the final DAG that consisted of 21 nodes, a potential causal pathway was represented by a green line connecting the exposure of tsDMARDs (green node with triangle) to the outcome of congenital anomaly (blue node with “I”). One mediator, “Maternal Medical History: Infection” was depicted as a blue node on the causal pathway from the exposure to the outcome, connected by unidirectional green arrows. We identified 1 factor (green node) as an ancestor of the exposure and 12 factors (blue nodes) as ancestors of the outcome. There were 6 factors identified as ancestors of both the exposure *and* outcome (red nodes), which form the biasing paths (red arrows) of this exposure-outcome relationship. One collider, where two or more arrowheads meet at a node, was identified, where maternal rheumatic “Disease activity” acts as a collider between maternal race/ethnicity and smoking.

A well-constructed DAG has practical implications for revealing factors that should be considered as confounders of the exposure-outcome relationship in a pharmacoepidemiologic study. Using DAGitty (11), our final DAG generated one minimally sufficient adjustment set to adjust for in our subsequent multivariable model [i.e., the lowest number of factors to consider as confounders that obtains an unbiased estimate of the total causal effect between exposure and outcome (41)], consisting of 6 factors: Maternal demographics of 1) age, 2) place of residence (urban/rural), 3) race/ethnicity; 4) SES, Concomitant medication of 5) csDMARDs; and 6) Maternal rheumatic disease activity.

## Discussion

The development of DAGs offers many benefits during the study design process of perinatal pharmacoepidemiologic studies. DAGs allow researchers to visualize causal pathways, detect biases and confounders, and reduce the risk of over- or under-adjustment to enhance the rigor and validity of studies (3, 4). After finalizing a DAG, minimally sufficient adjustment set(s) can be identified to inform robust analytical epidemiologic models. Nonetheless, consideration of other approaches to informing causal inference is helpful as DAGs represent only one of the complementary frameworks for defining, identifying, and estimating causal effects. For example, the counterfactual (potential outcomes) framework defines causal effects by comparing the outcomes an individual would experience under different exposure levels, even though only one of these outcomes is observable (42). While DAGs focus on visualizing assumed causal pathways, counterfactual-based approaches define causal effects by considering “what would have happened if” scenarios and provides a framework to estimate causal effects under hypothetical interventions. Our DAG-based approach also complements this framework by guiding confounder identification (42).

Even with a well-developed DAG that visualizes all potential and existing factors, operationalizing these factors into measurable variables requires careful consideration, as data availability in varying data sources, such as in administrative data, may present unique challenges and limitations. As pregnant individuals are often excluded from clinical trials, the generation of perinatal evidence must rely on observational data (1). Given that these are often rare exposures and outcomes, pharmacoepidemiologic studies evaluating the perinatal impact of novel drugs must leverage large, population-based datasets such as administrative health data. The development of our DAG serves as a prime example of how to operationalize factors from a DAG into administrative health data variables for the purposes of evaluating the impact of a novel drug on perinatal outcomes. From our minimally sufficient adjustment set of 6 factors [i.e., Maternal demographics of 1) age, 2) place of residence (urban/rural), 3) race/ethnicity; 4) SES, Concomitant medication of 5) csDMARDs; and 6) Maternal rheumatic disease activity], the following variables were identified to be available in administrative health data: tsDMARD exposure, congenital anomaly, maternal age and place of residence, and concomitant csDMARDs (Table 1). However, since administrative health data are not primarily gathered for research purposes, this limits the ability to operationalize certain factors, namely, SES, disease activity and race/ethnicity, which are not available as variables.

**Table 1:** Operationalization of factors identified for the DAG on tsDMARDs and congenital anomaly in administrative health data.

Variable^a^	Administrative health data	
	Availability	Example^b^
tsDMARDs	Yes	(1) DINs in BC Pharmanet database on prescription fills, or (2) ICD9 codes in the Medical Services Plan database on outpatient visits, or (3) ICD10 codes in the Discharge Abstract Database on hospitalizations in Population Data BC
Congenital anomaly	Yes	Variable in BC Perinatal Data Registry
Age	Yes	Consolidation file in Population Data BC
SES	No^c^	
Urban, rural	Yes	
Race/ethnicity	No	N/A
csDMARDs	Yes	DINs in BC Pharmanet database on prescription fills
Maternal rheumatic disease activity	No^c^	N/A

tsDMARDs, targeted synthetic disease-modifying antirheumatic drugs; DIN, drug identification number; ICD, International Classification of Diseases; SES, socioeconomic status; csDMARD, conventional synthetic disease-modifying antirheumatic drugs.

a The term *variable* is used here because we are operationalizing factors into measurable variables for inclusion in statistical analysis.

b In British Columbia (BC).

c May require the use of proxy variables.

For some factors, it may be feasible to utilize proxy variables. For example, rheumatic disease activity may be operationalized into variables related to healthcare utilization (i.e., outpatient visits, hospitalizations), radiographs ordered, and concomitant immunosuppressive drugs. SES may be measured using proxy variables such as neighborhood income quintile. Factors that cannot be operationalized will be excluded from the multivariable analyses, potentially leading to residual confounding, which should be acknowledged as a study limitation or addressed through methods for unmeasured confounders [e.g., imputation (43), propensity score calibration (44)]. We encourage researchers to adapt the operationalization of their DAG to reflect variables available in their own data sources and consider acknowledging the limitations or address them through other methods.

Our DAG demonstrates that clinical and methodological judgement, along with data availability, ultimately determines what is included in a multivariable model. In our example, although the DAGitty software identified “Disease activity” as part of the minimally sufficient adjustment set to be adjusted for, as previously identified from the DAG, this factor acts as a collider between race/ethnicity and smoking. However, since race/ethnicity and smoking are not variables available in administrative health data, they cannot be included in our multivariable model. In the absence of these two variables, we are unable to account for their biasing pathways, instead, we would now adjust for “Disease activity” in our model to minimize its confounding effect on the exposure and outcome.

While we have demonstrated the utility of DAGs in guiding confounder adjustments, there are inherent limitations that warrant discussion. Firstly, DAGs rely on the accuracy and completeness of assumed causal relationships between factors, as incorrect specification of causal relationships may lead to inappropriate adjustments and distorted effect estimates. In addition, the identification of causal relationships between factors are often based on literature, which may be influenced by publication bias. Therefore, DAGs are tools that provide clear visualizations of biasing pathways that can guide confounder selection.

By demonstrating the detailed process of designing a DAG based on a research question that captures the essence of perinatal pharmacoepidemiology, our study has highlighted many key considerations. Particularly, we highlight that when operationalizing factors into variables, the limitations of data sources such as administrative health data may alter the variables that are ultimately included in the multivariable model. However, DAGs offer an invaluable way to visualize biasing pathways and confounders in an ideal situation where all confounding variables are available to be adjusted for, resulting in increased robustness and validity of studies.

## Conclusion

Integrating DAGs into the design of pharmacoepidemiologic studies has benefits of visualizing complex causal relationships and biasing paths, making them valuable for evaluating perinatal impacts of medications. Detailing the development of our DAG provides particular value for future research in rheumatology, as disease activity and other factors associated with many rheumatic diseases complicate the relationships between drug exposure and perinatal outcomes. The nodes identified in our DAG and the DAG development process has been carefully designed with input from experts in the field, ensuring that relevant factors are thoughtfully selected or excluded as appropriate. Our DAG development process has implications for guiding future pharmacoepidemiologic studies by highlighting key factors that should be considered when examining drug exposure and perinatal outcomes. As the need for the perinatal impacts of emerging pharmacotherapies continues to grow, incorporating DAGs, such as the one presented in our paper, into pharmacoepidemiologic study designs will ultimately contribute to generating robust evidence to guide patient and provider decisions.

## Data Availability

The original contributions presented in the study are included in the article/Supplementary Material, further inquiries can be directed to the corresponding author.
