# Supplementary material for: Evidence-based directed acyclic graphs for perinatal pharmacoepidemiologic studies in rheumatology: a structured approach for development and implementation in administrative health data
Source: Front Epidemiol. 2026 Mar 10;6:1737016. doi: 10.3389/fepid.2026.1737016 (PMC13008904; doi:10.3389/fepid.2026.1737016)
Supplement: Supplementary file 1 [file Supplementaryfile1.docx]

dag {

" " [outcome,pos="0.698,-1.177"]

" " [exposure,pos="-1.747,-1.205"]

"Concomitant Rx: csDMARDs" [pos="-0.522,-0.440"]

"Disease activity" [pos="-0.803,0.094"]

"MD: Age" [pos="-0.530,-1.082"]

"MD: Place of residence (Urban/Rural)" [pos="-0.522,-0.650"]

"MD: Race/ethnicity" [pos="-0.611,-0.214"]

"MD: SES" [pos="-0.525,-0.863"]

"MMHx: BMI" [pos="0.512,-1.680"]

"MMHx: DM" [pos="0.551,-2.132"]

"MMHx: Gest DM" [pos="0.515,-1.908"]

"MMHx: Gest HTN" [pos="0.640,-2.357"]

"MMHx: Infection" [pos="-0.499,-1.439"]

"MMHx: Nutritional Deficiency" [pos="0.058,-2.280"]

"OHx: Parity ≥1" [pos="0.066,-1.562"]

"OHx: Prior pregnancy w/CA" [pos="0.050,-1.810"]

"PC: Background risk of CA" [pos="0.231,-0.352"]

"PC: Multifetal pregnancy" [pos="0.250,-0.576"]

"PEx: Assisted conception" [pos="0.278,-0.110"]

"PEx: Chemical, physical or biological hazard " [pos="0.337,0.111"]

"Prior Rx: cs/bDMARDs " [pos="-1.572,-1.473"]

"SHx: Alcohol/Substance" [pos="0.041,-2.058"]

"SHx: Smoking" [pos="-0.483,0.024"]

" " -> " "

" " -> "MMHx: Infection"

"Concomitant Rx: csDMARDs" -> " "

"Concomitant Rx: csDMARDs" -> " "

"Disease activity" -> " "

"Disease activity" -> " "

"MD: Age" -> " "

"MD: Age" -> " "

"MD: Place of residence (Urban/Rural)" -> " "

"MD: Place of residence (Urban/Rural)" -> " "

"MD: Race/ethnicity" -> " "

"MD: Race/ethnicity" -> " "

"MD: Race/ethnicity" -> "Disease activity"

"MD: SES" -> " "

"MD: SES" -> " "

"MMHx: BMI" -> " "

"MMHx: DM" -> " "

"MMHx: Gest DM" -> " "

"MMHx: Gest HTN" -> " "

"MMHx: Infection" -> " "

"MMHx: Nutritional Deficiency" -> " "

"OHx: Parity ≥1" -> " "

"OHx: Prior pregnancy w/CA" -> " "

"PC: Background risk of CA" -> " "

"PC: Multifetal pregnancy" -> " "

"PEx: Assisted conception" -> " "

"PEx: Chemical, physical or biological hazard " -> " "

"Prior Rx: cs/bDMARDs " -> " "

"SHx: Alcohol/Substance" -> " "

"SHx: Smoking" -> " "

"SHx: Smoking" -> "Disease activity"

}
