# Supplementary material for: Evidence-based directed acyclic graphs for perinatal pharmacoepidemiologic studies in rheumatology: a structured approach for development and implementation in administrative health data
Source: Front Epidemiol. 2026 Mar 10;6:1737016. doi: 10.3389/fepid.2026.1737016 (PMC13008904; doi:10.3389/fepid.2026.1737016)
Supplement: Supplementary file 2 [file Datasheet1.docx]

**
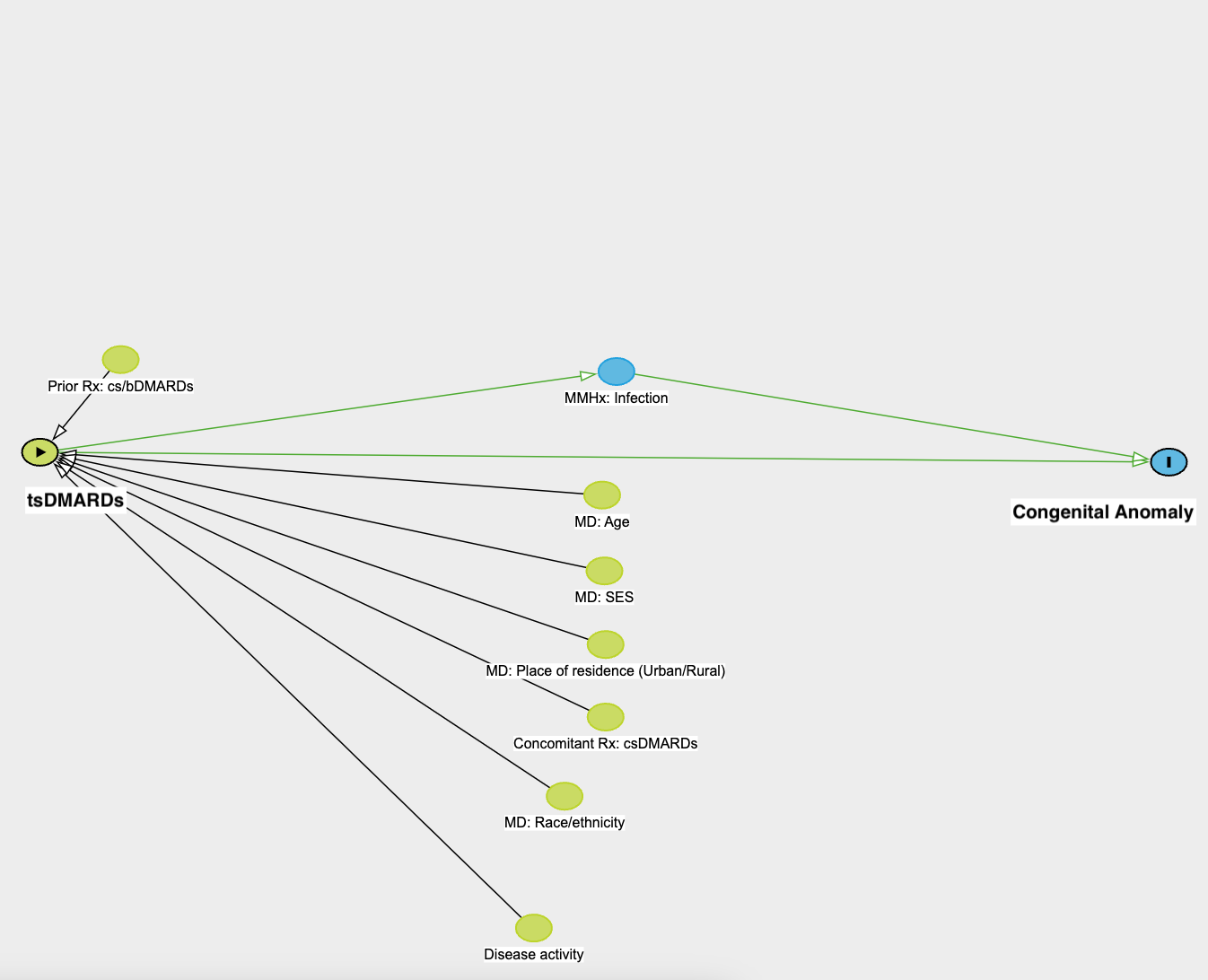
**

**Supplementary Figure 1.** Initial DAG development identifying factors affecting the exposure.

**
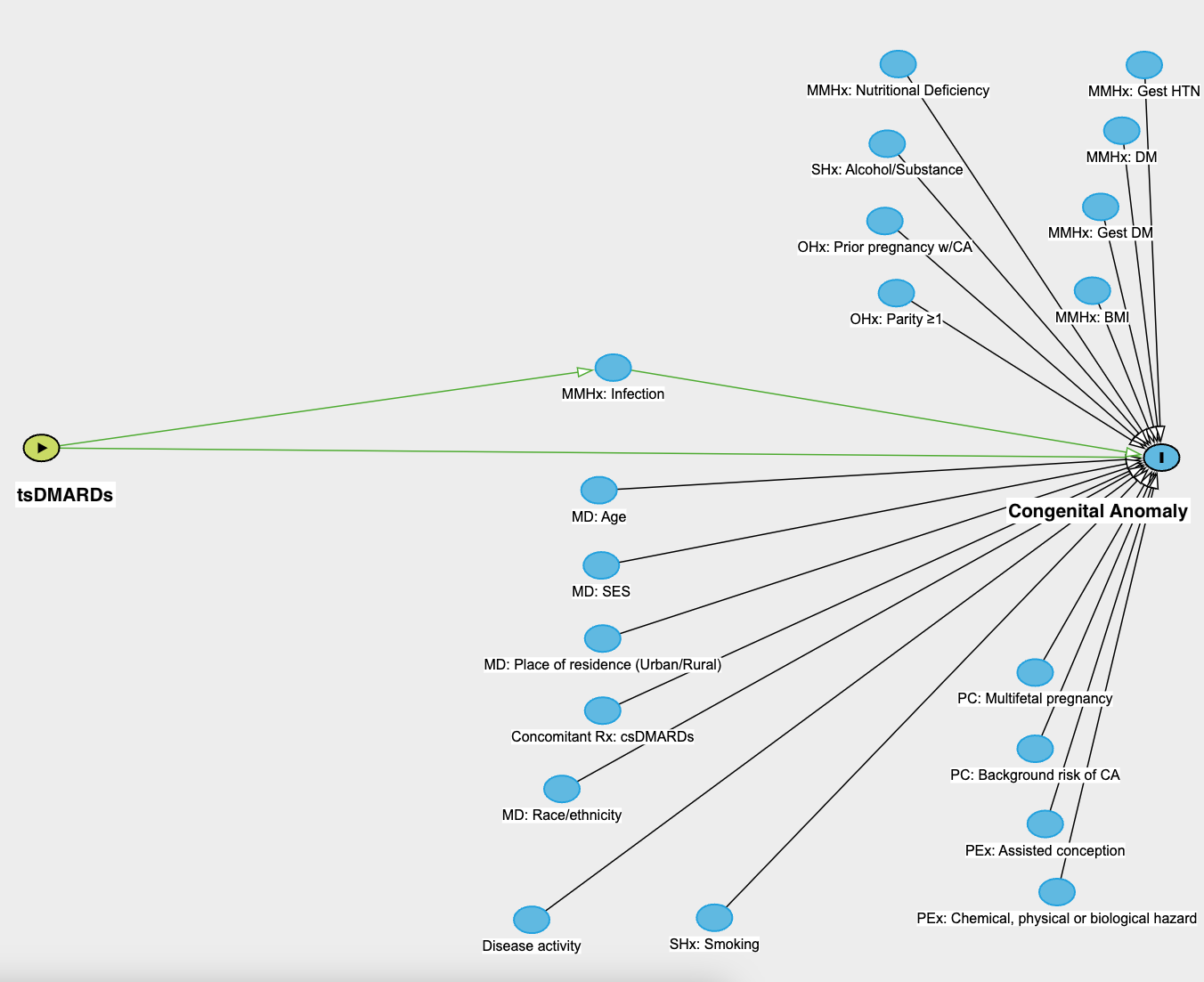
**

**Supplementary Figure 2.** Initial DAG development identifying factors affecting the outcome.
